# The unanti­cipated oxidation of a tertiary amine in a tetra­cyclic glyoxal-cyclam condensate yielding zinc(II) coordinated to a sterically hindered amine oxide

**DOI:** 10.1107/S2056989024001889

**Published:** 2024-03-06

**Authors:** Daniel J. Hubin, Blue M. Cunningham, Timothy J. Hubin, Jonathan P. Ebel, Jeanette A. Krause, Allen G. Oliver

**Affiliations:** a Department of Chemistry & Physics, Southwestern Oklahoma State University, Weatherford, OK 73096, USA; b Department of Chemistry, University of Cincinnati, Cincinnati, OH 45221, USA; c Department of Chemistry & Biochemistry, University of Notre Dame, Notre Dame, IN 46556, USA; University of Kentucky, USA

**Keywords:** crystal structure, zinc(II), glyoxal-cyclam

## Abstract

The complex, tri­chlorido­(1,4,11-tri­aza-8-azonia­tetra­cyclo­[6.6.2.0^4,16^.0^11,15^]hexa­decane 1-oxide-κ*O*)zinc(II) monohydrate, [ZnCl_3_(C_12_H_23_N_4_O)]·H_2_O, (**I**), has monoclinic symmetry (space group *P*2_1_/*n*) at 120 K. The zinc(II) center adopts a slightly distorted tetra­hedral coordination geometry and is coordinated by three chlorine atoms and the oxygen atom of the oxidized tertiary amine of the tetra­cycle. The amine nitro­gen atom, inside the ligand cleft, is protonated and forms a hydrogen bond to the oxygen of the amine oxide. Additional hydrogen-bonding inter­actions involve the protonated amine, the water solvate oxygen atom, and one of the chloro ligands.

## Abstract 2

The crystal structure of the first reported glyoxal–tetra­aza­macrocycle condensate amine oxide is presented. The sterically hindered oxidized amine binds zinc(II) through the oxygen atom in its folded cleft, with an inter­nal hydrogen bond across the cleft between the oxygen and a protonated tertiary nitro­gen.

## 1. Chemical context

Tetra­cyclic tetra­amines formed by the condensation of di­aldehyde glyoxal and tetra­aza­macrocycles, such as cyclen and cyclam, have been known since the 1980s (Alcock *et al.*, 1980 ▸; Weisman *et al.*, 1980 ▸). They can act as rigid, sterically hindered, bidentate chelates to form coordination complexes, but have not been exploited fully for this purpose (Hubin, McCormick, Busch & Alcock 1998 ▸; Hubin *et al.*, 1999 ▸, 2002 ▸; May *et al.*, 2004 ▸, Won *et al.*, 2015 ▸). Instead, their greatest utility has been as crucial starting materials for the synthesis of ethyl­ene cross-bridged tetra­aza­macrocycles (Weisman *et al.*, 1990 ▸, 1996 ▸; Wong *et al.*, 2000 ▸; Hubin, 2003 ▸; Matz *et al.*, 2015 ▸). Their folded structures generally lead to di­alkyl­ation of only two non-adjacent nitro­gens, which can then be reduced to ethyl­ene cross-bridged ligands whose rigid and topologically complex transition-metal complexes exhibit astounding kinetic stability under harsh conditions (Hubin, McCormick, Collinson, *et al.*, 1998 ▸; Hubin *et al.*, 2000 ▸, 2003 ▸; Sun *et al.*, 2002 ▸; Boswell *et al.*, 2004 ▸; Woodin *et al.*, 2005 ▸; Odendaal *et al.*, 2011 ▸; Matz *et al.*, 2015 ▸; Jones *et al.*, 2015 ▸).

Tertiary amines, like those found in glyoxal-tetra­aza­macrocycle condensates, are known to oxidize to amine oxides under oxidizing conditions, usually in the presence of hydrogen peroxide or 3-chloro­perbenzoic acid (O’Neal *et al.*, 2001 ▸; Bernier *et al.*, 2009 ▸). These amine oxides can be reduced back to amines in the presence of reducing agents (Hayashi *et al.*, 1959 ▸); zinc metal is often involved in the reduction reaction (Emerson & Rees, 1962 ▸; Kagami & Motoki, 1978 ▸; Jousseaume & Chanson, 1987 ▸; Balicki, 1989 ▸). In the present case, the tertiary amine was oxidized to the amine oxide in the presence of air, methanol, and zinc(II) chloride. Mol­ecular oxygen is able to oxidize tertiary amines (Bernier *et al.*, 2009 ▸), although it is generally inefficient and often is improved by the presence of transition metal ions, which may form metal-oxo catalysts *in situ*, although unlikely in the present case with zinc(II) (Jain & Sain, 2002 ▸; Wang *et al.*, 1999 ▸; Imada *et al.*, 2003 ▸). Under development in our labs are more efficient ways to make the mono- and di­amine oxides of glyoxal-tetra­aza­macrocycle condensates – at present both hydrogen peroxide and 3-chloro­perbenzoic acid have shown increased activity over mol­ecular oxygen – and will be reported in due course. 
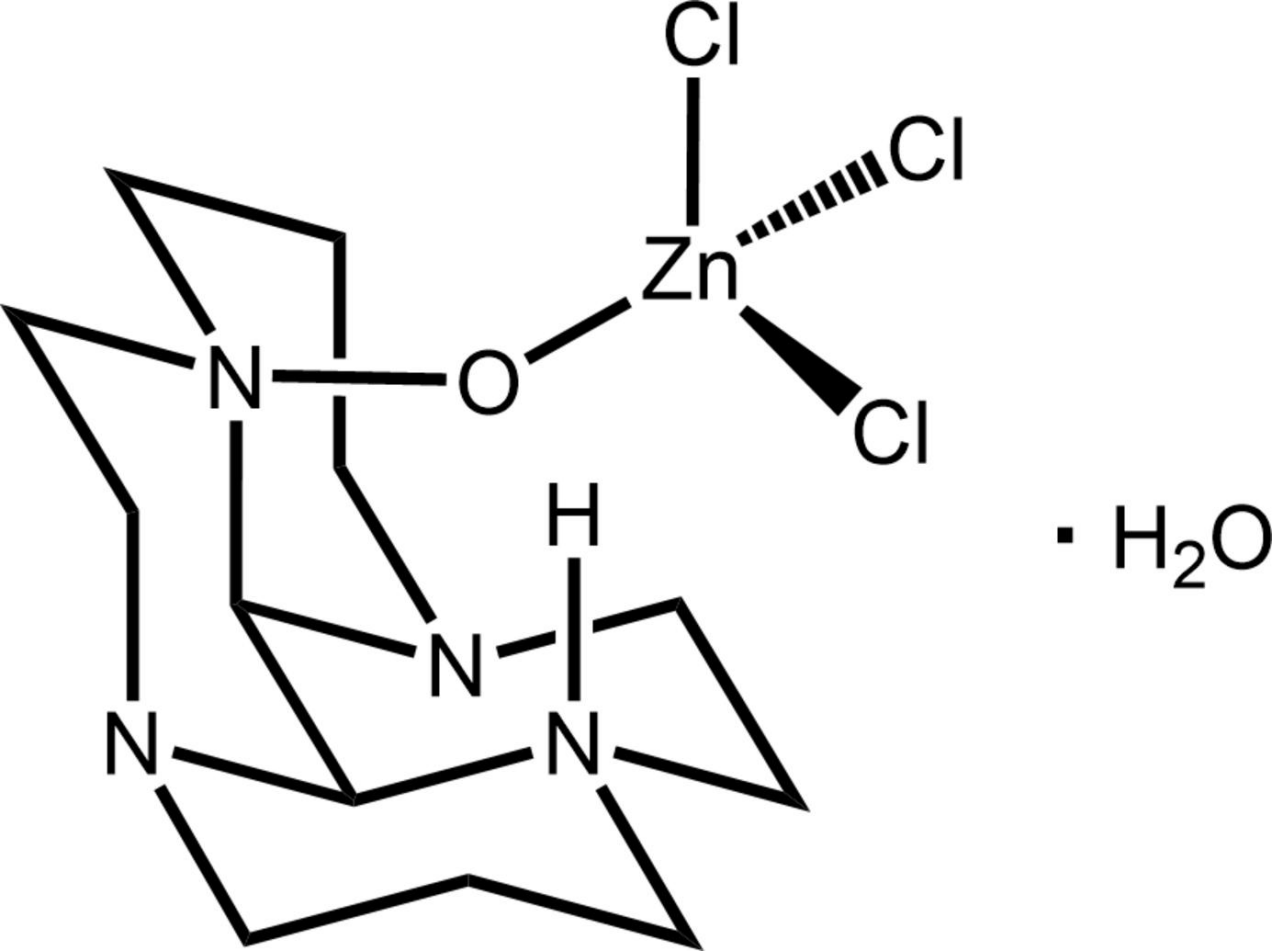


Here, we present the first example of a glyoxal-tetra­aza­macrocycle condensate that serendipitously oxidized to a mono-amine-oxide during a complexation reaction with zinc(II) chloride. The resulting sterically demanding amine oxide coordinates in a monodentate fashion to a zinc(II) ion concomitantly coordinated to three additional chloro ligands.

## 2. Structural commentary

**Figure 1:**
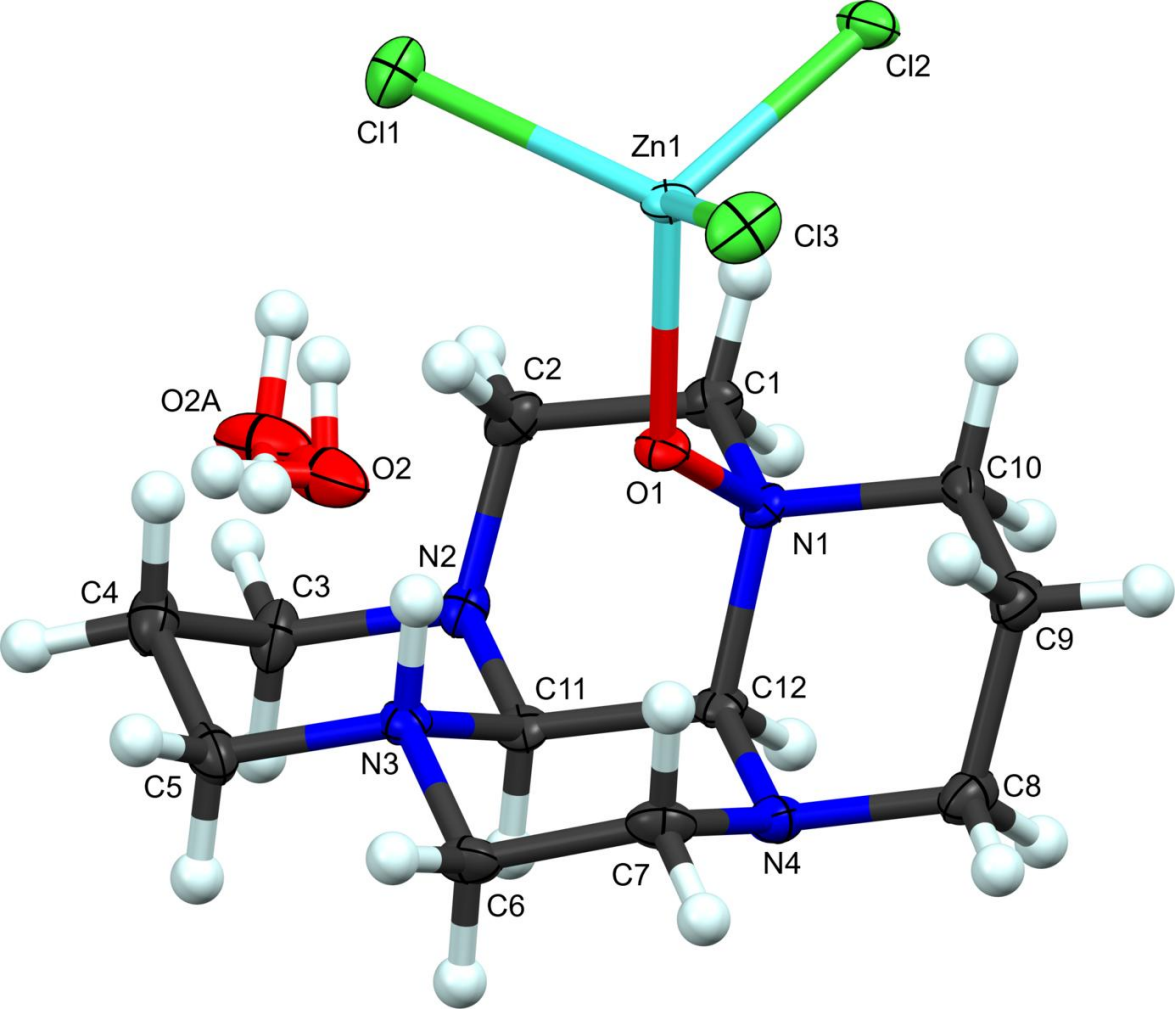
The mol­ecular structure of (**I**). Atomic displacement ellipsoids shown at 50% probability with hydrogen atoms shown as spheres of arbitrary radius.

We pioneered the use of glyoxal-tetra­aza­macrocycle condensates as rigid, bulky, bidentate ligands for transition metal ions (Hubin, McCormick, Busch & Alcock 1998 ▸), and have continued our efforts (Hubin *et al.*, 1999 ▸, 2002 ▸; May *et al.*, 2004 ▸, Won *et al.*, 2015 ▸) in coordinating various metal-containing species, in this case ZnCl_2_, to these bidentate amines of the glyoxal-cyclam condensate. During the course of the current work, air oxidation of one amine occurred, which produced an amine oxide moiety that subsequently resulted in coordination of [ZnCl_3_]^−^ in a monodentate fashion. In the majority of the unoxidized amine examples, where two non-adjacent amine nitro­gen atoms point into the cleft of the folded ligand, the metal ion coordinates in a bidentate fashion [palladium(II) and copper(II) examples: Hubin, McCormick, Alcock*, *et al.**, 1998; Hubin *et al.*, 2002 ▸; May *et al.*, 2004 ▸; Won *et al.*, 2015 ▸]. However, in the present case (Fig. 1 ▸), the oxygen atom, O1, extends the reach of the amine oxide and renders bidentate coord­ination unfavorable. Furthermore, the oxygen of the amine oxide moiety is situated in the center of the tetra­cycle cavity by virtue of the distorted tetra­hedral geometry about the atoms participating in the intra­molecular N3⋯O1 hydrogen-bonding inter­action. In addition, the oxygen atom fills most of this cavity created by the tetra­cycle, thus the larger than expected N1—O1—Zn1 bond angle [124.84 (9)°, Table 1 ▸] is instrumental in minimizing the steric hindrance caused by the bulk of the tri­chloro zinc unit. An inter­esting comparison can be drawn to our copper(I) glyoxal-cyclam condensate structure (Hubin *et al.*, 1999 ▸). In that case, the low coordination number preferred by copper(I), along with the steric bulk of the ligand, resulted in a copper(I) complex with two cyclam-glyoxal ligands coordinated in a linear fashion to the metal.

## 3. Supra­molecular features

**Figure 2:**
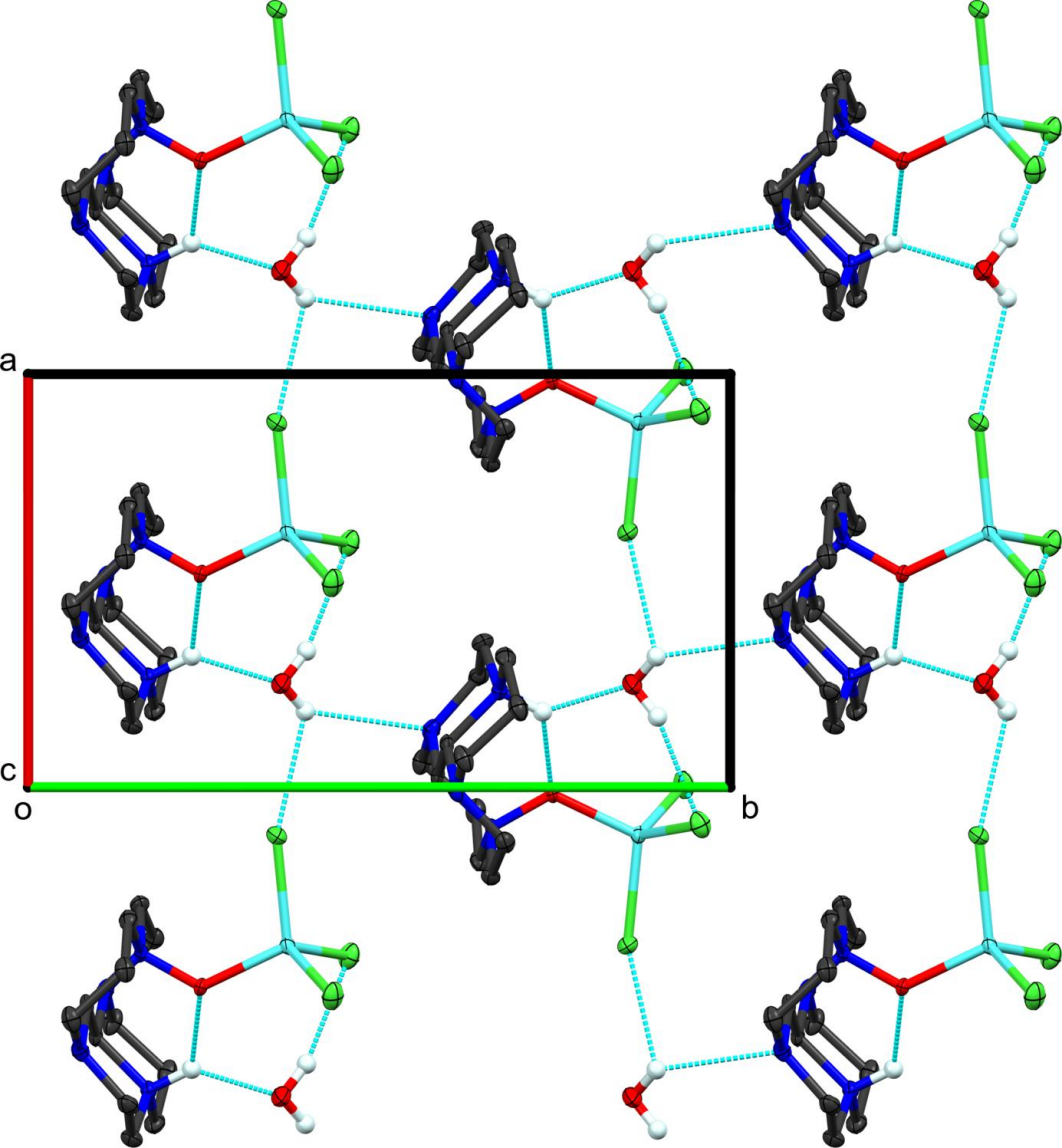
Packing diagram of (**I**) viewed along the *c*-axis. Hydrogen atoms, except those involved in hydrogen bonding, and one component of the disordered water of crystallization are omitted for clarity. Light-blue dashed lines represent hydrogen-bonding inter­actions. One layer of mol­ecules is shown for clarity.

Within the asymmetric unit, the ring nitro­gen atom, N1, forms a bifurcated hydrogen bond with both the water, O2, and *N*-oxide oxygen, O1, atoms (see Fig. 2 ▸, Table 2 ▸ for details). One water hydrogen forms a hydrogen bond to one chlorine, Cl1, of the standard mol­ecule resulting in an 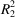 (10) ring (Etter *et al.*, 1990 ▸). The remaining water hydrogen atom forms a bifurcated hydrogen bond to a neighboring chlorine, Cl2, related by translation along the *a*-axis and to a tetra­cycle nitro­gen atom related by the screw-axis parallel to the *b*-axis. This is true for both components of the disordered water mol­ecule (see below). The overall motif is a di-periodic network (Nespolo, 2019 ▸) of hydrogen-bonded mol­ecules parallel to the *ab* plane. The remaining inter­actions within the structure (primarily C—H⋯Cl) are van der Waals contacts that direct the packing.

## 4. Database survey

We have found only two structural analogues of this zinc(II) coordination sphere – tertiary amine oxide and three chlorides coordinated to tetra­hedral zinc(II) (Jasiewicz *et al.*, 2011 ▸; refcodes: EWOZOG, EWOZUM). The Jasiewicz complexes utilize a spartein backbone ligand, which naturally form a folded structure with the amine lone pair of electrons pointed either concave or convex to the remainder of the structure. Analogous to our tetra­aza­macrocycle-glyoxal condensate, this generates either a concave or convex metal binding site. The most direct comparison to our own compound would be the concave isomer [(-)-spartein-16-ium *N*-1-oxide]tri­chloro­zinc(II). In both this complex and our own, an important non-covalent inter­action is the hydrogen bond formed by the oxygen of the amine oxide and the proton located on the non-adjacent nitro­gen. The folded nature of the amine oxide functionalized concave backbones allow the zinc atom to fit tightly inside the ligand. Notably, the sparteine compound shows similar average C—N—O bond angles (111.0° *cf* 110.68°) and a larger N—O—Zn bond angle than our compound [127.4 (5) and 124.84 (9)°, respectively]. These two angles work in conjunction to determine how far into the cavity the metal atom can approach. The smaller N—O—Zn angle of our tetra­aza­macrocyclic ligand allows the zinc to sit further into and, ideally, inter­act more strongly with atoms forming the cavity.

## 5. Synthesis and crystallization

The cyclam-glyoxal condensate was prepared according to a literature procedure (Le Baccon *et al.*, 2001 ▸): 0.24 g (1 mmol) of cyclam-glyoxal and 0.14 g (1 mmol) of ZnCl_2_ were stirred for three days in methanol (20 mL) in the presence of air. A white solid product precipitated and was filtered from the solution on a fine glass frit, and washed with a minimal amount of methanol before being dried under vacuum. X-ray quality colorless block crystals were obtained by ether diffusion into a 2-butanone solution.

## 6. Refinement

The structure was solved by dual-space methods (*SHELXT;* Sheldrick, 2015*a* ▸) and refinement was routine (*SHELXL;* Sheldrick, 2015*b* ▸; Table 3 ▸). All non-hydrogen atoms were refined with anisotropic atomic displacement parameters. The water of crystallization exhibited mild positional disorder that was modeled over two equal occupancy sites. Hydrogen atoms on the water and protonated amine nitro­gen were initially located in a difference-Fourier map. The amine hydrogen atom was ultimately refined using a riding model. The coord­inates of the water hydrogen atoms were allowed to refine, with similarity restraints applied to all four O—H distances. Atomic displacement parameters of these hydrogen atoms were tied to that of the N or O to which they are bonded. All other hydrogen atoms were positioned at geometrically calculated positions with C—H = 0.99 or 1.00 Å for methyl­ene and methine carbon atoms respectively; *U*_iso_(H) = 1.5 × *U*_eq_(O) or 1.2 × *U*_eq_(N/C).

## Supplementary Material

Crystal structure: contains datablock(s) I. DOI: 10.1107/S2056989024001889/pk2704sup1.cif

Structure factors: contains datablock(s) I. DOI: 10.1107/S2056989024001889/pk2704Isup2.hkl

CCDC reference: 2335501

Additional supporting information:  crystallographic information; 3D view; checkCIF report

## Figures and Tables

**Table 1:** Selected geometric parameters (Å, °)

Zn1—O1	1.9856 (11)	Zn1—Cl1	2.2616 (5)
Zn1—Cl3	2.2345 (5)	Zn1—Cl2	2.2856 (5)
			
O1—Zn1—Cl3	107.03 (4)	Cl3—Zn1—Cl2	111.244 (19)
O1—Zn1—Cl1	109.33 (4)	Cl1—Zn1—Cl2	106.531 (18)
Cl3—Zn1—Cl1	111.812 (19)	N1—O1—Zn1	124.84 (9)
O1—Zn1—Cl2	110.93 (3)		

**Table 2:** Hydrogen-bond geometry (Å, °)

*D*—H⋯*A*	*D*—H	H⋯*A*	*D*⋯*A*	*D*—H⋯*A*
N3—H3⋯O1	1.00	2.15	2.8297 (18)	123
N3—H3⋯O2	1.00	1.94	2.763 (6)	138
N3—H3⋯O2*A*	1.00	1.98	2.786 (7)	136
O2—H2*C*⋯Cl2^i^	0.87 (4)	2.61 (8)	3.154 (6)	121 (7)
O2—H2*C*⋯N4^ii^	0.87 (4)	2.61 (8)	3.262 (6)	133 (8)
O2—H2*D*⋯Cl1	0.87 (4)	2.39 (4)	3.227 (6)	162 (6)
O2*A*—H2*E*⋯Cl2^i^	0.87 (4)	2.69 (8)	3.295 (6)	128 (9)
O2*A*—H2*E*⋯N4^ii^	0.87 (4)	2.84 (9)	3.202 (6)	107 (7)
O2*A*—H2*F*⋯Cl1	0.98 (6)	2.04 (7)	2.996 (6)	163 (5)

Symmetry codes: (i) 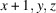 ; (ii) 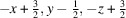 .

**Table 3:** Experimental details

Crystal data	
Chemical formula	[ZnCl_3_(C_12_H_23_N_4_O)]·H_2_O
*M*_r_	429.08
Crystal system, space group	Monoclinic, *P*2_1_/*n*
Temperature (K)	120
*a*, *b*, *c* (Å)	8.7253 (8), 14.3482 (14), 14.0718 (13)
β (°)	104.760 (2)
*V* (Å^3^)	1703.5 (3)
*Z*	4
Radiation type	Mo *K*α
μ (mm^−1^)	1.92
Crystal size (mm)	0.21 × 0.12 × 0.09

Data collection	
Diffractometer	Bruker APEXII CCD
Absorption correction	Numerical (*SADABS*; Krause *et al.*, 2015 ▸)
*T*_min_, *T*_max_	0.699, 0.793
No. of measured, independent and observed [*I* > 2σ(*I*)] reflections	32386, 4237, 3572
*R*_int_	0.044
(sin θ/λ)_max_ (Å^−1^)	0.667

Refinement	
*R*[*F* ^2^ > 2σ(*F* ^2^)], *wR*(*F* ^2^), *S*	0.024, 0.056, 1.01
No. of reflections	4237
No. of parameters	220
No. of restraints	3
H-atom treatment	H atoms treated by a mixture of independent and constrained refinement
Δρ_max_, Δρ_min_ (e Å^−3^)	0.42, −0.33

Computer programs: *APEX4* and *SAINT* (Bruker, 2021 ▸), *SHELXT2014/5* (Sheldrick, 2015*a* ▸), *SHELXL2019/2* (Sheldrick, 2015*b* ▸), *OLEX2 1.5* (Dolomanov *et al.*, 2009 ▸), *Mercury* (Macrae *et al.*, 2020 ▸), and *publCIF* (Westrip, 2010 ▸).

## supplementary crystallographic information

### Crystal data

**Table:**

[ZnCl_3_(C_12_H_23_N_4_O)]·H_2_O	*F*(000) = 888
*M**_r_* = 429.08	*D*_x_ = 1.673 Mg m^−^^3^
Monoclinic, *P*2_1_/*n*	Mo *K*α radiation, λ = 0.71073 Å
*a* = 8.7253 (8) Å	Cell parameters from 8725 reflections
*b* = 14.3482 (14) Å	θ = 2.5–28.1°
*c* = 14.0718 (13) Å	µ = 1.92 mm^−^^1^
β = 104.760 (2)°	*T* = 120 K
*V* = 1703.5 (3) Å^3^	Block, colorless
*Z* = 4	0.21 × 0.12 × 0.09 mm

### Data collection

**Table:**

Bruker APEXII CCD diffractometer	4237 independent reflections
Radiation source: X-ray	3572 reflections with *I* > 2σ(*I*)
Detector resolution: 8.33 pixels mm^-1^	*R*_int_ = 0.044
φ and ω scans	θ_max_ = 28.3°, θ_min_ = 2.1°
Absorption correction: numerical (SADABS; Krause *et al.*, 2015)	*h* = −11→11
*T*_min_ = 0.699, *T*_max_ = 0.793	*k* = −19→19
32386 measured reflections	*l* = −18→18

### Refinement

**Table:**

Refinement on *F*^2^	Primary atom site location: dual
Least-squares matrix: full	Secondary atom site location: difference Fourier map
*R*[*F*^2^ > 2σ(*F*^2^)] = 0.024	Hydrogen site location: mixed
*wR*(*F*^2^) = 0.056	H atoms treated by a mixture of independent and constrained refinement
*S* = 1.01	*w* = 1/[σ^2^(*F*_o_^2^) + (0.0244*P*)^2^ + 0.8924*P*] where *P* = (*F*_o_^2^ + 2*F*_c_^2^)/3
4237 reflections	(Δ/σ)_max_ = 0.002
220 parameters	Δρ_max_ = 0.42 e Å^−^^3^
3 restraints	Δρ_min_ = −0.33 e Å^−^^3^

### Special details

**Table:**

Geometry. All esds (except the esd in the dihedral angle between two l.s. planes) are estimated using the full covariance matrix. The cell esds are taken into account individually in the estimation of esds in distances, angles and torsion angles; correlations between esds in cell parameters are only used when they are defined by crystal symmetry. An approximate (isotropic) treatment of cell esds is used for estimating esds involving l.s. planes.

### Fractional atomic coordinates and isotropic or equivalent isotropic displacement parameters (Å^2^)

**Table:**

	*x*	*y*	*z*	*U*_iso_*/*U*_eq_	Occ. (<1)
Zn1	0.38352 (2)	0.63073 (2)	0.74182 (2)	0.01361 (6)	
Cl1	0.40927 (6)	0.54285 (3)	0.61336 (3)	0.02305 (10)	
Cl2	0.11765 (5)	0.64610 (3)	0.72669 (3)	0.02054 (9)	
Cl3	0.50553 (6)	0.56614 (3)	0.88570 (3)	0.02517 (10)	
O1	0.48379 (13)	0.75415 (7)	0.73622 (8)	0.0143 (2)	
N1	0.40264 (16)	0.83922 (9)	0.72215 (10)	0.0128 (3)	
N2	0.49127 (17)	0.88977 (9)	0.54778 (10)	0.0158 (3)	
N3	0.73297 (16)	0.82844 (9)	0.66434 (10)	0.0150 (3)	
H3	0.682588	0.767367	0.672505	0.018*	
N4	0.63893 (17)	0.92560 (10)	0.81784 (10)	0.0168 (3)	
C1	0.2840 (2)	0.84123 (12)	0.62400 (12)	0.0168 (3)	
H1A	0.200438	0.794152	0.622381	0.020*	
H1B	0.233240	0.903358	0.612818	0.020*	
C2	0.3667 (2)	0.82071 (12)	0.54517 (12)	0.0187 (4)	
H2A	0.413817	0.757581	0.555104	0.022*	
H2B	0.289180	0.822203	0.480114	0.022*	
C3	0.5688 (2)	0.87961 (12)	0.46713 (13)	0.0211 (4)	
H3A	0.620783	0.939150	0.458245	0.025*	
H3B	0.487426	0.866169	0.405469	0.025*	
C4	0.6919 (2)	0.80197 (13)	0.48621 (13)	0.0221 (4)	
H4A	0.639472	0.741237	0.489173	0.027*	
H4B	0.745027	0.799462	0.431914	0.027*	
C5	0.8131 (2)	0.81999 (13)	0.58233 (13)	0.0210 (4)	
H5A	0.890506	0.768148	0.596338	0.025*	
H5B	0.871458	0.878211	0.577319	0.025*	
C6	0.8502 (2)	0.85260 (12)	0.75866 (13)	0.0200 (4)	
H6A	0.900038	0.913375	0.751790	0.024*	
H6B	0.934682	0.804743	0.773996	0.024*	
C7	0.7694 (2)	0.85777 (12)	0.84095 (13)	0.0203 (4)	
H7A	0.727310	0.795572	0.851407	0.024*	
H7B	0.847102	0.876364	0.902397	0.024*	
C8	0.5621 (2)	0.93873 (12)	0.89888 (12)	0.0206 (4)	
H8A	0.507952	0.999931	0.891293	0.025*	
H8B	0.644199	0.939192	0.962106	0.025*	
C9	0.4420 (2)	0.86239 (12)	0.90143 (12)	0.0198 (4)	
H9A	0.496925	0.801698	0.916110	0.024*	
H9B	0.388001	0.875842	0.953828	0.024*	
C10	0.3211 (2)	0.85768 (11)	0.80278 (12)	0.0171 (3)	
H10A	0.244040	0.807317	0.803837	0.020*	
H10B	0.262410	0.917304	0.789926	0.020*	
C11	0.6037 (2)	0.90373 (11)	0.63997 (12)	0.0145 (3)	
H11	0.659039	0.963672	0.634126	0.017*	
C12	0.5264 (2)	0.91649 (11)	0.72469 (12)	0.0142 (3)	
H12	0.466658	0.976619	0.712488	0.017*	
O2	0.7488 (7)	0.6396 (4)	0.7042 (5)	0.0387 (13)	0.5
H2C	0.816 (8)	0.605 (6)	0.684 (5)	0.058*	0.5
H2D	0.670 (6)	0.604 (4)	0.677 (5)	0.058*	0.5
O2A	0.7280 (7)	0.6345 (4)	0.6550 (4)	0.0399 (14)	0.5
H2E	0.806 (8)	0.618 (7)	0.704 (5)	0.060*	0.5
H2F	0.631 (8)	0.598 (4)	0.631 (4)	0.060*	0.5

### Atomic displacement parameters (Å^2^)

**Table:**

	*U*^11^	*U*^22^	*U*^33^	*U*^12^	*U*^13^	*U*^23^
Zn1	0.01529 (10)	0.00965 (9)	0.01544 (10)	−0.00118 (7)	0.00307 (7)	0.00082 (7)
Cl1	0.0301 (2)	0.0209 (2)	0.0180 (2)	0.00448 (18)	0.00584 (17)	−0.00187 (16)
Cl2	0.0149 (2)	0.01714 (19)	0.0298 (2)	−0.00291 (15)	0.00600 (17)	−0.00108 (16)
Cl3	0.0342 (3)	0.0201 (2)	0.0177 (2)	0.00302 (18)	0.00018 (18)	0.00485 (16)
O1	0.0134 (6)	0.0079 (5)	0.0209 (6)	0.0019 (4)	0.0034 (5)	0.0021 (4)
N1	0.0146 (7)	0.0085 (6)	0.0155 (7)	0.0017 (5)	0.0040 (5)	0.0003 (5)
N2	0.0217 (8)	0.0133 (7)	0.0132 (7)	0.0028 (5)	0.0055 (6)	0.0009 (5)
N3	0.0149 (7)	0.0112 (6)	0.0193 (7)	−0.0003 (5)	0.0050 (6)	0.0010 (5)
N4	0.0191 (7)	0.0156 (7)	0.0148 (7)	−0.0036 (6)	0.0026 (6)	−0.0011 (5)
C1	0.0145 (8)	0.0153 (8)	0.0180 (8)	0.0013 (6)	−0.0004 (6)	0.0008 (6)
C2	0.0208 (9)	0.0169 (8)	0.0160 (8)	0.0017 (7)	0.0006 (7)	−0.0013 (6)
C3	0.0313 (10)	0.0180 (8)	0.0168 (8)	0.0072 (7)	0.0113 (7)	0.0018 (7)
C4	0.0293 (10)	0.0181 (8)	0.0219 (9)	0.0053 (7)	0.0117 (8)	0.0007 (7)
C5	0.0206 (9)	0.0182 (8)	0.0280 (9)	0.0021 (7)	0.0133 (8)	0.0011 (7)
C6	0.0140 (8)	0.0199 (8)	0.0244 (9)	−0.0035 (7)	0.0015 (7)	−0.0009 (7)
C7	0.0171 (9)	0.0206 (9)	0.0202 (8)	−0.0039 (7)	−0.0010 (7)	0.0009 (7)
C8	0.0297 (10)	0.0175 (8)	0.0149 (8)	−0.0040 (7)	0.0060 (7)	−0.0019 (7)
C9	0.0272 (10)	0.0172 (8)	0.0169 (8)	−0.0015 (7)	0.0089 (7)	−0.0001 (7)
C10	0.0186 (9)	0.0141 (8)	0.0217 (8)	0.0015 (7)	0.0110 (7)	−0.0011 (7)
C11	0.0181 (8)	0.0083 (7)	0.0176 (8)	0.0003 (6)	0.0052 (7)	0.0024 (6)
C12	0.0179 (8)	0.0080 (7)	0.0168 (8)	−0.0021 (6)	0.0048 (6)	0.0006 (6)
O2	0.020 (2)	0.0159 (18)	0.084 (4)	0.0026 (16)	0.019 (3)	0.000 (3)
O2A	0.018 (2)	0.0196 (19)	0.079 (4)	0.0014 (15)	0.007 (3)	0.003 (3)

### Geometric parameters (Å, º)

**Table:**

Zn1—O1	1.9856 (11)	C4—C5	1.512 (3)
Zn1—Cl3	2.2345 (5)	C4—H4A	0.9900
Zn1—Cl1	2.2616 (5)	C4—H4B	0.9900
Zn1—Cl2	2.2856 (5)	C5—H5A	0.9900
O1—N1	1.3996 (16)	C5—H5B	0.9900
N1—C1	1.500 (2)	C6—C7	1.504 (3)
N1—C10	1.508 (2)	C6—H6A	0.9900
N1—C12	1.542 (2)	C6—H6B	0.9900
N2—C11	1.427 (2)	C7—H7A	0.9900
N2—C2	1.465 (2)	C7—H7B	0.9900
N2—C3	1.469 (2)	C8—C9	1.522 (2)
N3—C6	1.495 (2)	C8—H8A	0.9900
N3—C5	1.499 (2)	C8—H8B	0.9900
N3—C11	1.536 (2)	C9—C10	1.516 (2)
N3—H3	1.0000	C9—H9A	0.9900
N4—C12	1.429 (2)	C9—H9B	0.9900
N4—C7	1.470 (2)	C10—H10A	0.9900
N4—C8	1.475 (2)	C10—H10B	0.9900
C1—C2	1.498 (2)	C11—C12	1.523 (2)
C1—H1A	0.9900	C11—H11	1.0000
C1—H1B	0.9900	C12—H12	1.0000
C2—H2A	0.9900	O2—H2C	0.87 (4)
C2—H2B	0.9900	O2—H2D	0.87 (4)
C3—C4	1.523 (2)	O2A—H2E	0.87 (4)
C3—H3A	0.9900	O2A—H2F	0.98 (6)
C3—H3B	0.9900		
			
O1—Zn1—Cl3	107.03 (4)	N3—C5—H5A	109.6
O1—Zn1—Cl1	109.33 (4)	C4—C5—H5A	109.6
Cl3—Zn1—Cl1	111.812 (19)	N3—C5—H5B	109.6
O1—Zn1—Cl2	110.93 (3)	C4—C5—H5B	109.6
Cl3—Zn1—Cl2	111.244 (19)	H5A—C5—H5B	108.1
Cl1—Zn1—Cl2	106.531 (18)	N3—C6—C7	110.23 (14)
N1—O1—Zn1	124.84 (9)	N3—C6—H6A	109.6
O1—N1—C1	110.64 (12)	C7—C6—H6A	109.6
O1—N1—C10	111.42 (12)	N3—C6—H6B	109.6
C1—N1—C10	110.01 (13)	C7—C6—H6B	109.6
O1—N1—C12	107.32 (11)	H6A—C6—H6B	108.1
C1—N1—C12	108.81 (12)	N4—C7—C6	110.56 (14)
C10—N1—C12	108.55 (12)	N4—C7—H7A	109.5
C11—N2—C2	116.85 (13)	C6—C7—H7A	109.5
C11—N2—C3	111.79 (14)	N4—C7—H7B	109.5
C2—N2—C3	113.48 (14)	C6—C7—H7B	109.5
C6—N3—C5	110.74 (13)	H7A—C7—H7B	108.1
C6—N3—C11	109.42 (13)	N4—C8—C9	112.18 (14)
C5—N3—C11	110.19 (12)	N4—C8—H8A	109.2
C6—N3—H3	108.8	C9—C8—H8A	109.2
C5—N3—H3	108.8	N4—C8—H8B	109.2
C11—N3—H3	108.8	C9—C8—H8B	109.2
C12—N4—C7	117.04 (13)	H8A—C8—H8B	107.9
C12—N4—C8	112.30 (14)	C10—C9—C8	109.44 (14)
C7—N4—C8	113.11 (14)	C10—C9—H9A	109.8
C2—C1—N1	109.18 (13)	C8—C9—H9A	109.8
C2—C1—H1A	109.8	C10—C9—H9B	109.8
N1—C1—H1A	109.8	C8—C9—H9B	109.8
C2—C1—H1B	109.8	H9A—C9—H9B	108.2
N1—C1—H1B	109.8	N1—C10—C9	110.21 (14)
H1A—C1—H1B	108.3	N1—C10—H10A	109.6
N2—C2—C1	110.04 (14)	C9—C10—H10A	109.6
N2—C2—H2A	109.7	N1—C10—H10B	109.6
C1—C2—H2A	109.7	C9—C10—H10B	109.6
N2—C2—H2B	109.7	H10A—C10—H10B	108.1
C1—C2—H2B	109.7	N2—C11—C12	112.94 (14)
H2A—C2—H2B	108.2	N2—C11—N3	113.56 (13)
N2—C3—C4	112.29 (14)	C12—C11—N3	110.86 (13)
N2—C3—H3A	109.1	N2—C11—H11	106.3
C4—C3—H3A	109.1	C12—C11—H11	106.3
N2—C3—H3B	109.1	N3—C11—H11	106.3
C4—C3—H3B	109.1	N4—C12—C11	113.04 (14)
H3A—C3—H3B	107.9	N4—C12—N1	113.60 (13)
C5—C4—C3	109.50 (15)	C11—C12—N1	110.04 (13)
C5—C4—H4A	109.8	N4—C12—H12	106.5
C3—C4—H4A	109.8	C11—C12—H12	106.5
C5—C4—H4B	109.8	N1—C12—H12	106.5
C3—C4—H4B	109.8	H2C—O2—H2D	92 (7)
H4A—C4—H4B	108.2	H2E—O2A—H2F	124 (9)
N3—C5—C4	110.20 (14)		
			
Zn1—O1—N1—C1	−62.44 (15)	C12—N1—C10—C9	−57.12 (16)
Zn1—O1—N1—C10	60.28 (15)	C8—C9—C10—N1	58.25 (18)
Zn1—O1—N1—C12	178.98 (9)	C2—N2—C11—C12	−48.08 (19)
O1—N1—C1—C2	−55.24 (16)	C3—N2—C11—C12	178.82 (13)
C10—N1—C1—C2	−178.78 (13)	C2—N2—C11—N3	79.25 (17)
C12—N1—C1—C2	62.43 (16)	C3—N2—C11—N3	−53.85 (17)
C11—N2—C2—C1	52.82 (19)	C6—N3—C11—N2	176.31 (13)
C3—N2—C2—C1	−174.84 (14)	C5—N3—C11—N2	54.33 (18)
N1—C1—C2—N2	−59.05 (17)	C6—N3—C11—C12	−55.28 (17)
C11—N2—C3—C4	55.33 (19)	C5—N3—C11—C12	−177.27 (13)
C2—N2—C3—C4	−79.42 (19)	C7—N4—C12—C11	−47.23 (19)
N2—C3—C4—C5	−56.6 (2)	C8—N4—C12—C11	179.49 (13)
C6—N3—C5—C4	−176.21 (14)	C7—N4—C12—N1	79.08 (18)
C11—N3—C5—C4	−55.01 (17)	C8—N4—C12—N1	−54.20 (18)
C3—C4—C5—N3	56.74 (19)	N2—C11—C12—N4	176.91 (13)
C5—N3—C6—C7	−178.14 (14)	N3—C11—C12—N4	48.17 (18)
C11—N3—C6—C7	60.20 (17)	N2—C11—C12—N1	48.73 (17)
C12—N4—C7—C6	51.16 (19)	N3—C11—C12—N1	−80.01 (16)
C8—N4—C7—C6	−175.92 (14)	O1—N1—C12—N4	−64.71 (16)
N3—C6—C7—N4	−56.85 (18)	C1—N1—C12—N4	175.53 (13)
C12—N4—C8—C9	54.02 (19)	C10—N1—C12—N4	55.82 (17)
C7—N4—C8—C9	−81.15 (18)	O1—N1—C12—C11	63.16 (15)
N4—C8—C9—C10	−55.69 (19)	C1—N1—C12—C11	−56.59 (16)
O1—N1—C10—C9	60.84 (16)	C10—N1—C12—C11	−176.31 (13)
C1—N1—C10—C9	−176.07 (13)		

### Hydrogen-bond geometry (Å, º)

**Table:**

*D*—H···*A*	*D*—H	H···*A*	*D*···*A*	*D*—H···*A*
N3—H3···O1	1.00	2.15	2.8297 (18)	123
N3—H3···O2	1.00	1.94	2.763 (6)	138
N3—H3···O2*A*	1.00	1.98	2.786 (7)	136
O2—H2*C*···Cl2^i^	0.87 (4)	2.61 (8)	3.154 (6)	121 (7)
O2—H2*C*···N4^ii^	0.87 (4)	2.61 (8)	3.262 (6)	133 (8)
O2—H2*D*···Cl1	0.87 (4)	2.39 (4)	3.227 (6)	162 (6)
O2*A*—H2*E*···Cl2^i^	0.87 (4)	2.69 (8)	3.295 (6)	128 (9)
O2*A*—H2*E*···N4^ii^	0.87 (4)	2.84 (9)	3.202 (6)	107 (7)
O2*A*—H2*F*···Cl1	0.98 (6)	2.04 (7)	2.996 (6)	163 (5)
C1—H1*A*···Cl2	0.99	2.78	3.6198 (18)	143
C2—H2*B*···Cl3^iii^	0.99	2.96	3.7412 (18)	136
C3—H3*A*···N2^iv^	0.99	2.64	3.348 (2)	129
C4—H4*B*···Cl2^v^	0.99	2.92	3.6201 (19)	128
C5—H5*A*···Cl2^i^	0.99	2.92	3.8290 (19)	154
C5—H5*B*···Cl3^vi^	0.99	2.90	3.8493 (19)	161
C6—H6*A*···Cl1^vi^	0.99	2.87	3.6295 (18)	135
C6—H6*B*···Cl2^i^	0.99	2.95	3.8679 (19)	154
C7—H7*A*···O1	0.99	2.40	2.953 (2)	115
C8—H8*A*···Cl2^vii^	0.99	2.73	3.6088 (19)	149
C8—H8*B*···Cl1^viii^	0.99	2.73	3.7009 (19)	168
C10—H10*A*···Cl2	0.99	2.67	3.5420 (17)	147
C10—H10*B*···Cl1^vii^	0.99	2.89	3.7018 (17)	139
C12—H12···Cl2^vii^	1.00	2.74	3.6524 (17)	152

Symmetry codes: (i) *x*+1, *y*, *z*; (ii) −*x*+3/2, *y*−1/2, −*z*+3/2; (iii) *x*−1/2, −*y*+3/2, *z*−1/2; (iv) −*x*+1, −*y*+2, −*z*+1; (v) *x*+1/2, −*y*+3/2, *z*−1/2; (vi) −*x*+3/2, *y*+1/2, −*z*+3/2; (vii) −*x*+1/2, *y*+1/2, −*z*+3/2; (viii) *x*+1/2, −*y*+3/2, *z*+1/2.
